# Early and Later Life Stress Alter Brain Activity and Sleep in Rats

**DOI:** 10.1371/journal.pone.0069923

**Published:** 2013-07-26

**Authors:** Jelena Mrdalj, Ståle Pallesen, Anne Marita Milde, Finn Konow Jellestad, Robert Murison, Reidun Ursin, Bjørn Bjorvatn, Janne Grønli

**Affiliations:** 1 Department of Biological and Medical Psychology, University of Bergen, Bergen, Norway; 2 Norwegian Competence Center for Sleep Disorders, Haukeland University Hospital, Bergen, Norway; 3 Department of Psychosocial Science, University of Bergen, Bergen, Norway; 4 Department of Biomedicine, University of Bergen, Bergen, Norway; 5 Department of Global Public Health and Primary Care, University of Bergen, Bergen, Norway; Hôpital du Sacré-Coeur de Montréal, Canada

## Abstract

Exposure to early life stress may profoundly influence the developing brain in lasting ways. Neuropsychiatric disorders associated with early life adversity may involve neural changes reflected in EEG power as a measure of brain activity and disturbed sleep. The main aim of the present study was for the first time to characterize possible changes in adult EEG power after postnatal maternal separation in rats. Furthermore, in the same animals, we investigated how EEG power and sleep architecture were affected after exposure to a chronic mild stress protocol. During postnatal day 2–14 male rats were exposed to either long maternal separation (180 min) or brief maternal separation (10 min). Long maternally separated offspring showed a sleep-wake nonspecific reduction in adult EEG power at the frontal EEG derivation compared to the brief maternally separated group. The quality of slow wave sleep differed as the long maternally separated group showed lower delta power in the frontal-frontal EEG and a slower reduction of the sleep pressure. Exposure to chronic mild stress led to a lower EEG power in both groups. Chronic exposure to mild stressors affected sleep differently in the two groups of maternal separation. Long maternally separated offspring showed more total sleep time, more episodes of rapid eye movement sleep and higher percentage of non-rapid eye movement episodes ending in rapid eye movement sleep compared to brief maternal separation. Chronic stress affected similarly other sleep parameters and flattened the sleep homeostasis curves in all offspring. The results confirm that early environmental conditions modulate the brain functioning in a long-lasting way.

## Introduction

Across the lifespan, a cascade of neurobiological changes occurs, the most significant taking place during early life. Exposure to early life stress may shape the developing central nervous system and profoundly influence major brain reorganization despite its plasticity. Such impact may be reflected in neural measures, like altered brain activity measured by electroencephalographic (EEG) power. Reduced EEG power has been observed in post-institutionalized children compared to family-raised children [1] and in adults exposed to early life stress compared to non-exposed controls [2]. Furthermore, early stressful experiences have been linked to altered mental health later in life, like mood and anxiety disorders and even neurodegenerative disorders such as dementia [3], [4]. One suggested explanation has been increased sensitivity for stress [3]. For instance, stress-altered cortical processing has been linked to negative childhood experiences [5].

If neural changes after early life stress result in reduction of EEG power in childhood and in adulthood, the occurrence should be sleep-wake nonspecific. According to the synaptic homeostasis hypothesis, being awake usually results in synaptic potentiation, whereas sleep is a period where the brain downscales its synaptic activation [6]. However, recent data indicate that sleep may also be involved in strengthening synaptic transmission [7]. Sleep is a fundamental process and is essential for many physiological and psychological functions. However, disturbances of sleep are widespread in victims of adverse childhood experiences. These often report insomnia, increased nightly awakenings and restless sleep [8]–[10]. To date, no sleep EEG recording studies have been conducted in these victims. The preclinical literature describes some changes in sleep architecture after early environmental factors. Although the studies are scarce and the findings are divergent, long maternal separation (LMS) in rats is reported to change total sleep time (decrease or increase) and increase wakefulness compared to non-handled, handled and brief maternally separated (BMS) conditions [11]–[13].

In depressed patients, sleep EEG shows a reduction of the restorative slow wave activity during deep slow wave sleep (SWS) [14]–[16], as well as an increased high-frequency activity across sleep stages [17] compared to healthy controls. Such changes may reflect markers of a vulnerability to stress and psychiatric disorders. Borbely and colleagues have suggested that sleep regulation and sleep homeostasis are impaired in patients with depression [14]. This hypothesis is based on the two-process model of sleep regulation where the need for sleep (the homeostatic process) accumulates during wakefulness [18]. The best characterized physiological marker of sleep pressure is the level of slow wave activity (the low range delta EEG power) during SWS. The slow wave activity is high early in the sleep period and decreases progressively to reach low levels in late sleep [18], [19]. Whether early and later life stress and possibly altered brain activation has an impact on sleep homeostasis remains to be investigated.

Long maternal separation and low maternal care in non-human primates and rodents have been shown to induce several neuronal changes [20]–[30]. However, no previous study has investigated brain activity using spectral analysis of EEG frequencies following maternal separation in rats. Against this backdrop, we conducted a study to investigate how maternal separation would influence rat EEG: 1) during the course of the total sleep period, 2) on sleep and wake-specific frequencies from frontal and frontal-parietal derivations and 3) sleep homeostasis. In addition, we examined changes in sleep architecture. Furthermore, we investigated how maternal separation in rats affects the response to a chronic mild stress (CMS) regime in adulthood. It is well known that CMS induces depression-like symptoms, including typical sleep changes and neurobiological changes [25], [31]–[34]. Considering that maternal separation may be predictive of stress responsiveness later in life, we specifically aimed to examine whether LMS predisposes offspring to be more susceptible to CMS in terms of more reduced brain activity, impaired sleep homeostasis and more disturbed sleep than BMS.

## Methods

### Ethical Approval

This study was approved by the Norwegian Animal Research Authority (Permit Number: 07/9421–2007025). The experiment was conducted in accordance with Norwegian laws and regulations controlling experiments in live animals. Norway has signed and ratified The European Convention for the protection of Vertebrate Animals used for Experimental and other Scientific Purposes, March 18, 1986. Surgical procedures were performed under hypnorm-dormicum anaesthesia, and every effort was made to minimize suffering of the animals.

### Experimental Subjects and Design

Outbred rats (Wistar, NTac:WH,Taconic, Denmark) were housed in an individually ventilated cage system (IVC system, Tecniplast®, Italy) comprising type III (425 x 266 x 185 mm, floor area 800 cm^2^) and type IV (480 x 375 x 210 mm, floor area 1400 cm^2^) cages at an ambient temperature of 22±1°C and air humidity of 52±2%. A controlled 12 h light/12 h dark schedule was maintained including 1 h gradual increase with lights fully on at 07∶00 A.M. Standard rodent diet (Rat and Mouse nr. 1 (RM1), Special Diets Services, Witham, Essex, England) and fresh water were available *ad libitum*, except when animals were exposed to food or water deprivation during the CMS procedure. For breeding, the rodent diet RM 3 was used. Bedding (Bee Kay Bedding, Scanbur BK) was changed once a week, except during postnatal days (PND) 0–14 and during the CMS protocol. The same personnel handled the animals throughout the study and gloves were used at all times.

**Figure 1:**
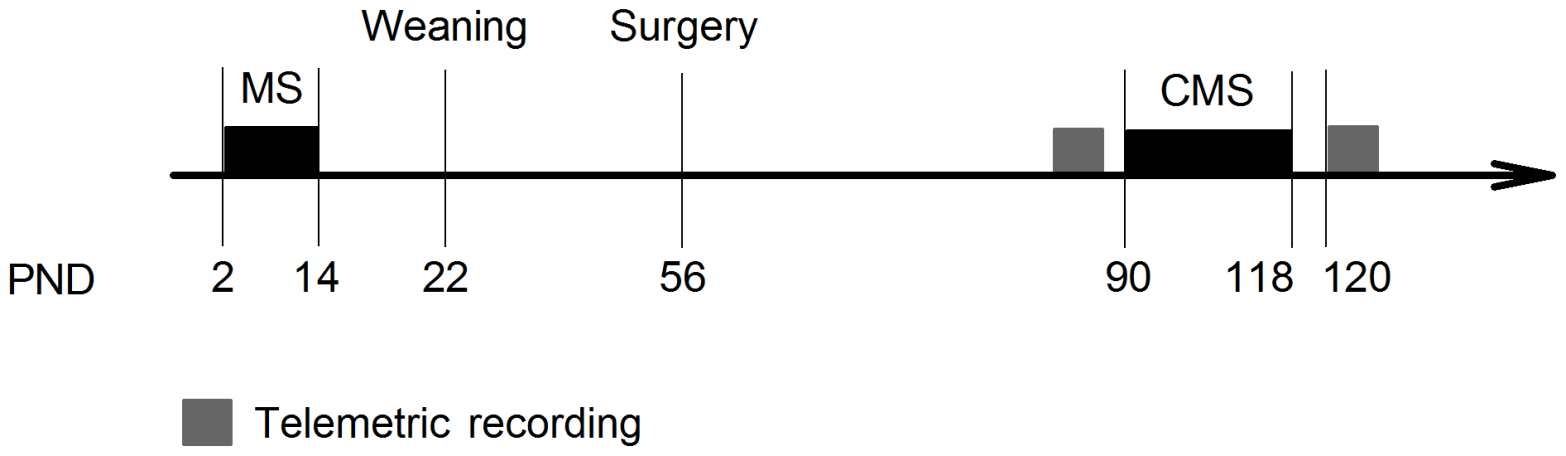
Experimental design. PND = postnatal day, MS = maternal separation, CMS = chronic mild stress. Telemetric recording: 12 h EEG/EMG recording during the inactive phase pre and post CMS.

An overview of the design is provided in Figure 1. New-born rats were daily exposed to either BMS of 10 min or LMS of 3 h, during PND 2–14. Weaning took place at PND 22, at which point offspring were separated by gender and group housed in IVC type IV cages. The present study includes 8 LMS and 8 BMS male offspring implanted with telemetric devices when 9 weeks old, and then housed individually in IVC type III cages. Exposure to 4 weeks of CMS started at PND 90. Sleep EEG was analysed in the rats’ 12 h inactive phase (07∶00 A.M. –07∶00 P.M.) pre and post CMS. The rats were not exposed to any stressor for at least 24 h before post CMS recordings. Blood samples for analysis of serum levels of corticosterone were taken 2–4 days prior to pre CMS recording. These data will be addressed in a separate paper including additional data.

### Stress conditions

#### Maternal separation (MS)

Sixteen female and 10 male rats were mated in our animal facility. To avoid possible effects of variation in litter size, cross-fostering procedures were performed within the first 24 h after birth. After cross-fostering each mother had 12 offspring, and was randomly assigned to one of the following protocols [28] during PND 2–14:

#### LMS group

Dams and their offspring were separated from each other once a day for a period of 180 min starting at 09∶00 A.M. The dam was first removed from its home cage, placed in a type III cage with food and water *ad libitum* and moved to a room adjacent to the offspring. The whole litter was then moved to a cage with cardboard divided compartments, one for each litter. Ambient temperature was regulated artificially by a heating lamp and monitored (PND 2–7∶32–34°C, PND 8–14∶28–30°C). The same cages and compartments were used every day and none of the cages were cleaned during the procedure. At the end of each daily separation dams and their offspring were reunited in reverse order.

#### BMS group

Dams and their offspring were separated from each other once a day for a period of 10 min starting at 09∶00 A.M. The same procedures were followed as for the LMS group except that the artificial heating was not provided.

### Chronic Mild Stress (CMS)

The CMS procedure was modified from Willner et al. [35] and Grønli et al. [36]. New stressors included were exposure to a new cage without bedding for 21 h, followed by 3 h exposure to 3 cm of water in cage. Otherwise, each of the 4 weeks of CMS consisted of one exposure to food deprivation (18 h) followed by restricted access to food (1 h); two periods of water deprivation (16 h and 20 h) followed by 1 h exposure to an empty water bottle; two periods with tilted cage (3 h); one exposure to social stress (paired caging, 2 h); one exposure to wet bedding (20 h); and one continuous light period of 36 h. The stressors were presented both during the rats’ active and inactive phase. See Table 1 for time and duration of each stressor applied. A one-hour sucrose consumption test was performed once a week preceded by 4 h of food and water deprivation. Data related to this will be published elsewhere.

**Table 1:** Chronic mild stress protocol.

Stressor	Monday	Tuesday	Wednesday	Thursday	Friday	Saturday	Sunday
Sucrose consumption test			16∶00→17∶00				
Food deprive	09∶00		12∶00→16∶00				15∶00→
Restricted food	09∶00→10∶00						
Water deprive			12∶00→16∶00, 17∶00→	10∶00	13∶00→	10∶00	
Empty bottle				09∶00→10∶00		09∶00→10∶00	
Tilted cage		10∶00→13∶00		10∶00→13∶00			
Paired caging	13∶00→15∶00						
Wet cage			17∶00→	13∶00			
New cage no bedding				13∶00→	10∶00		
Water in cage					10∶00→13∶00		
Bedding in cage					13∶00		
Continuous light						07∶00→	19∶00

### Surgery

For continuous measurement of EEG and electromyogram (EMG) data, a wireless transmitter system (4ET, Physiotel®; Data Sciences International) was implanted subcutaneously (s.c.). Each rat was anaesthetized with s.c. injection of a mixture of fentanyl 0.277 mg/kg animal, fluanizone 8.8 mg/kg, and midazolam 2.5 mg/kg (Hypnorm, Janssen; Dormicum, Roche), placed in a stereotaxic apparatus (Kopf®, USA) with the head fixed with incisor bars. The effect of anaesthesia was monitored during the whole procedure and additional anaesthesia was given at approximately 45 min intervals. A vertical incision was made over the dorsomedial lumbar region, where a sterile telemetric device (sensor and battery) was placed in an s.c. pocket. Vertical incision was made on the skull, and biopotential leads were s.c. tunnelled up to the neck region for the EEG and EMG derivations. Two leads were attached to the neck muscle for EMG recordings. Four leads were placed epidurally for frontal-frontal (FF) and frontal-parietal (FP) EEG recordings. Frontal leads were located 2 mm anterior to bregma and 2 mm lateral to the midline and the parietal lead was located 2 mm anterior to lambda and 2 mm lateral to the midline. The EEG leads were secured to the skull with dental acrylic (GC RELINE, America INC.) and the skin was closed with interrupted mattress sutures.

Postoperatively animals received buprenorphinum for analgesic purposes (Temgesic, Reckitt & Colman), 0.10 ml s.c. twice a day for 3 days. A minimum of 14 days was allowed for animals to recover and regain their preoperative weight. One day before surgery and during the first two postoperative days, antibiotics (Bactrim, Roche; 5 ml in 250 ml drinking water) were administered. One animal from the BMS group died before data collection due to postoperative complications.

### Telemetric Recording

The wireless recording device was calibrated to record signals in the range −1.25 to +1.25 mV. The transmitter was switched on by passing a magnet along the animal’s side at the site of the implanted battery. During recording all animals remained undisturbed in their home cages. EEG and EMG signals were collected continuously at a sampling rate of 250 Hz. Analogue band-pass filters of 1 Hz (high pass filter) and 100 Hz (low pass filter) were used. No additional filtering was used. Telemetry signals were collected through receiver type RPC-2 (Data Sciences International) placed directly beneath the home cage and connected to a data exchange matrix where signals were converted and transferred to the acquisition software Dataquest ART (version 4.1, Data Sciences International).

### EEG/EMG Analysis

#### Sleep staging

Scoring of sleep stages and power spectrum analysis was performed offline with Neuroscore software (version 2.0.1, Data Sciences International). Sleep stages were scored by use of FF-EEG signal filtered with high-pass at 3 Hz and low pass frequency at 35 Hz, and FP-EEG signal filtered with high-pass frequency at 0.5 Hz and low pass frequency at 35 Hz. The EMG signal was high-pass filtered at 5 Hz. A 50 Hz power line filter was applied to all channels. A semi-automatic scoring algorithm was applied on the filtered signals for 10 s epochs. Analysis thresholds (delta-ratio, theta-ratio, EMG-threshold, activity-threshold) were adjusted individually for each animal. REM sleep and wakefulness were manually re-scored due to missing criteria for muscle atonia in the automatic scoring algorithm for REM sleep. Sleep stages were defined according to Neckelmann and Ursin [37]: wakefulness was scored when there was high-frequency low voltage activity in EEG channels, high to moderate activity in EMG and activity threshold >0.1 cpm (counts per min); slow wave sleep 1 (SWS1; comparable to light sleep in humans) was scored when spindle activity (11–16 Hz) was present in the FF channel,<50% of delta (0.5–4 Hz) activity in the FP channel, and EMG reduced compared to wakefulness; slow wave sleep 2 (SWS2; comparable to deep sleep in humans) was scored with spindle activity present in FF and ≥50% of delta activity in FP channel and EMG activity equal to or lower than SWS1; REM sleep was scored when there was predominantly theta (6–9 Hz) activity in the FP channel and EMG activity was reduced to its lowest or abolished (muscle atonia). An inter-rater reliability between manual and semi-automatic scoring was evaluated in 6 randomly selected 12 h-recordings (a total of 4320 10 s epochs) yielding a satisfactory mean kappa of 0.73±0.03. The % agreement was 92.7% for wakefulness, 96.5% for REM sleep, 85.5% for SWS1 and 92.2% for SWS2, respectively.

The following sleep parameters were calculated: total sleep time; time spent in wakefulness, SWS1, SWS2 and REM sleep and for characterization of NREM sleep (SWS1+ SWS2) the number of episodes was calculated. For characterization of REM sleep we calculated number of episodes and mean episode duration and assessed the percentage of NREM episodes ending in REM sleep. For characterization of wakefulness we calculated the number of episodes, mean duration of episodes in addition to duration of the longest wakefulness episode.

### EEG Power Spectrum Analysis

For the investigation of EEG power spectrum distributions, an offline Fast Fourier Transform (FFT) analysis was applied on unfiltered EEG signals. Analysis was performed on 10 s intervals with Hamming Window and 75% overlap in FF and FP derivation separately. Total power in a range from 0.5–60 Hz was calculated without distinguishing between sleep stages. For each sleep stage the EEG power of the frequency bands characteristic for the stage was calculated: SWS (SWS1+ SWS2): delta (0.5–4.4 Hz) and theta (5.5–9.4 Hz) band; REM sleep: the theta, beta (19.5–34.4 Hz) and gamma (lower range, 35–60 Hz) band; wakefulness: beta and the gamma band. An average of different frequency bands was calculated from artefact-free 10 s epochs during the 12 h recording period. Mean values were used for statistical analysis. Removal of artefacts was performed in two steps: 1) manually: both EEG signals were visually inspected and all epochs containing movement or electrical artefacts were excluded; and 2) semi-automatically: all epochs with EEG power values exceeding mean value multiplied by a factor of 4 were visually inspected and removed if the high value was due to an artefact. For investigation of the homeostatic sleep regulation an average of low range delta (0.5–2.4 Hz) power during SWS was calculated from the FP derivation for each hour after lights on at 07∶00 A.M. FP derivation yields optimal recording of slow waves and was therefore chosen [37].

### Statistics

Statistica (version 8.0 or 10.0, StatSoft, Inc.) was used for all statistical analyses. Significance was set to *p*≤0.05 two-tailed. All significant overall effects of analysis of variance (ANOVA) were further tested with Fisher LSD post-hoc test. The homogeneity of variance was tested using Levene’s test. For all analyses "group" was used as the independent factor. To describe the impact of our data, we used Cohen’s d (d = (M1–M2)/SD pooled) to estimate the effect sizes for between group comparisons. An effect size of 0.2 to 0.3 is considered to be small, around 0.5 to be a medium effect and 0.8 and above, a large effect [38]. Positive d values represent increase and negative values represent decrease in the LMS compared to the BMS group.

EEG power densities for representative frequency bands in wakefulness, SWS and REM sleep as well as total EEG power, were analysed with ANOVA for repeated measures with “group” as independent factor and “recording day” as repeated measure.

Sleep homeostasis: To compare the homeostatic sleep pressure between groups, the mean of the low-range delta power at h 1 (07∶00–08∶00 A.M.) was analysed with a *t*-test for independent samples. To test the reduction in homeostatic sleep pressure during the 12 h sleep period, low-range delta power during the first 3 h of sleep (h 1–3, mean) was compared to the second 3 h period (h 4–6, mean) and to the third 3 h period (h 7–9, mean) of sleep with repeated measure ANOVA (“group×hour”). Due to the low amount of sleep in the fourth 3 h period (h 10–12), it was excluded from the analysis. Repeated measure ANOVA was also performed for each 3 h period with “group” as independent factor and “recording day” as repeated measure.

Sleep parameters: Repeated measure ANOVA was used with “group” as independent factor and “recording day” and “sleep stage” as repeated measures. Total sleep time and each of the parameters for characterization of REM sleep and wakefulness were analysed with ANOVA for repeated measures using “group” and “recording day”.

All data were acquired from 8 LMS and 7 BMS animals. Different degrees of freedom in the results section are due to exclusion of some animals from analysis because of poor quality in EEG signals, or values showing more than 2 standard deviations from the mean. The excluded animals also showed abnormal recovery in the postoperative log (e.g. infection, prolonged wound healing).

## Results

### Long Maternal Separation Lowered the Frontal EEG Power Across Sleep Stages

**Figure 2:**
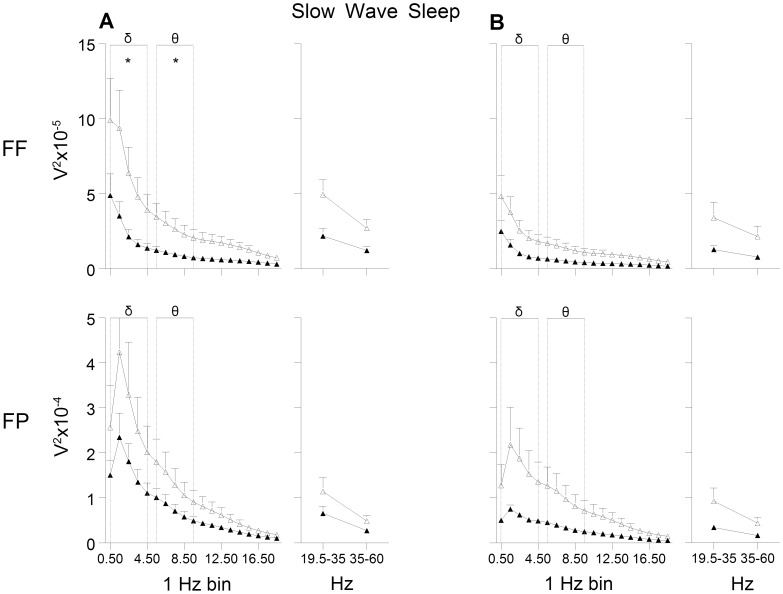
EEG power during slow wave sleep. (A) pre and (B) post exposure to chronic mild stress. FF = frontal-frontal derivation, FP = frontal-parietal derivation. Open triangles indicate brief maternally separated rats and filled triangles long maternally separated rats. The curves connect the mean values and SEM for each 1 Hz bin (from 0.5 to 19.4 Hz) and beta (19.5–35.4 Hz) and gamma band (35–60 Hz). Delta (δ) and theta (θ) bands were used for statistical analyses. FF: delta: group and recording day effect (F’s_(1,11)_ ≥4.8, *p≤*0.05); theta: group and recording day effect (F’s_(1,10)_≥5.4, *p*<0.05); FP: delta and theta: recording day effect (F’s_(1,12)_≥17.4, *p<*0.01).* p<0.05.

**Figure 3:**
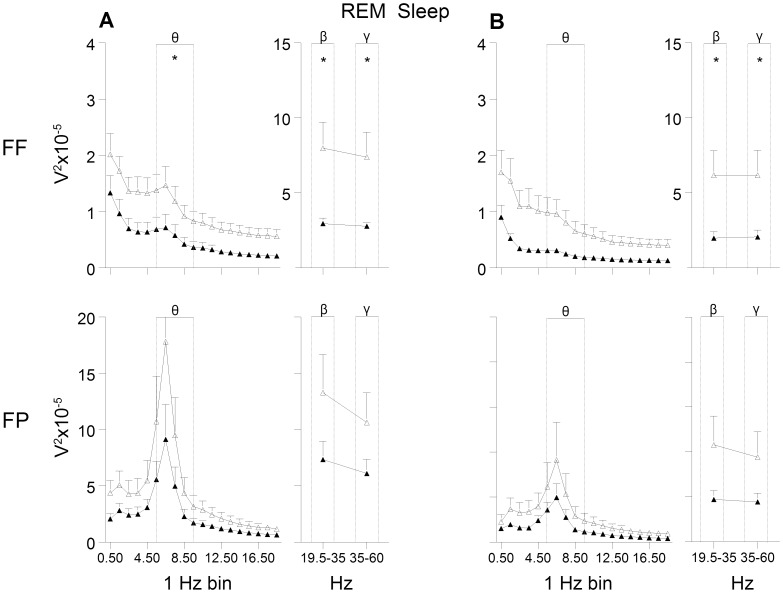
EEG power during rapid eye movement (REM) sleep. (A) pre and (B) post exposure to chronic mild stress. FF = frontal-frontal derivation, FP = frontal-parietal derivation. Open triangles indicate brief maternally separated rats and filled triangles long maternally separated rats. The curves connect the mean values and SEM for each 1 Hz bin (from 0.5 to 19.4 Hz) and beta (β) and gamma (γ) band. Theta (θ), beta and gamma bands were used for statistical analyses. FF: all bands: group and recording day effect (F’s_(1,11)_≥6.0, *p<*0.05). FP: recording day effect in beta, gamma (F’s_(1,12)_≥18.8, *p<*0.001) and theta (F_(1,11)_ = 12.9, *p<*0.01).* p<0.05.

**Figure 4:**
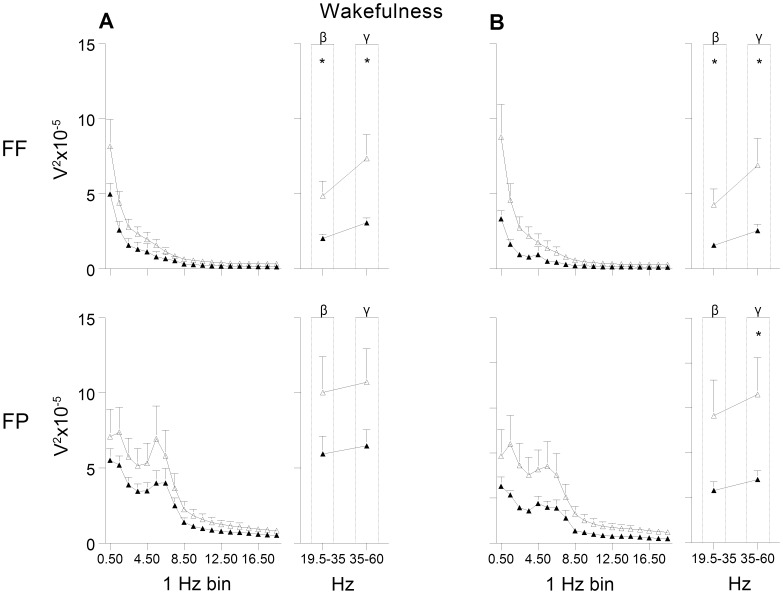
EEG power during wakefulness. (A) pre and (B) post exposure to chronic mild stress. FF = frontal-frontal derivation, FP = frontal-parietal derivation. Open triangles indicate brief maternally separated rats and filled triangles long maternally separated rats. The curves connect the mean values and SEM for each 1 Hz bin (from 0.5 to 19.4 Hz) and beta (β) and gamma (γ) band. Beta and gamma bands were used for statistical analyses. FF: group and recording day effect in beta (F’s_(1,12)_≥6.0, *p<*0.05), and gamma (F’s_(1,11)_≥5.8, *p<*0.05); FP: recording day effect in beta and gamma (F’s_(1,12)_≥8.3, *p<*0.05) and tendency for group effect in gamma (F_(1,12)_ = 4.1, *p* = 0.06).* p<0.05.

The general shape of the EEG power curves as a measure of brain activity was similar in the LMS and BMS offspring, but a generally dampened EEG power was observed in the LMS group, in the FF derivation in particular (see Figures 2–4).

A decrease was observed in total EEG power (0.5–60 Hz) in the FF derivation during the 12 h recording of the rats’ inactive phase (*p*<0.05; d = -1.46). Here, the EEG power in the LMS group was significantly lower compared to the BMS group in the frequency bands characteristic for wakefulness and the different sleep stages. The power of the delta band, the main characteristic frequency band of SWS, and the theta band was lower in the LMS group (Figure 2A). Furthermore, the most prominent frequencies for REM sleep, the theta band and the higher frequencies, were significantly lower compared to the BMS group (Figure 3A). In wakefulness the characteristic beta and gamma band were lower in LMS compared to BMS group (Figure 4A).

No effects were observed for the FP derivation.

All comparisons showed large effect sizes with negative values (d ranging from −0.7 to −1.9).

### Exposure to CMS in Adulthood Lowered EEG Power in All Offspring

In general, exposure to CMS in adulthood led to a parallel reduction of EEG power in the two groups in the FF derivation. LMS offspring decreased their total EEG power with 44.5% (±13.3%) and BMS offspring with 37.0% (±28.8%) (data not shown).

In the FP derivation, the LMS offspring showed significantly lower power in the gamma band in wakefulness (Figure 4B).

Most of the significant differences between LMS and BMS offspring observed in the FF derivation pre CMS were also present post CMS. LMS showed lower total EEG power and lower power of higher frequencies characteristic for REM sleep (Figure 3B) and wakefulness (Figure 4B) compared to BMS. There was a tendency for lower power of the theta band during REM sleep in the LMS group (p = 0.06, d =  −1.6).

All comparisons showed large effect sizes with negative values (d ranging from −0.6 to −1.6).

### Slower Reduction of Sleep Pressure in LMS Offspring

**Figure 5:**
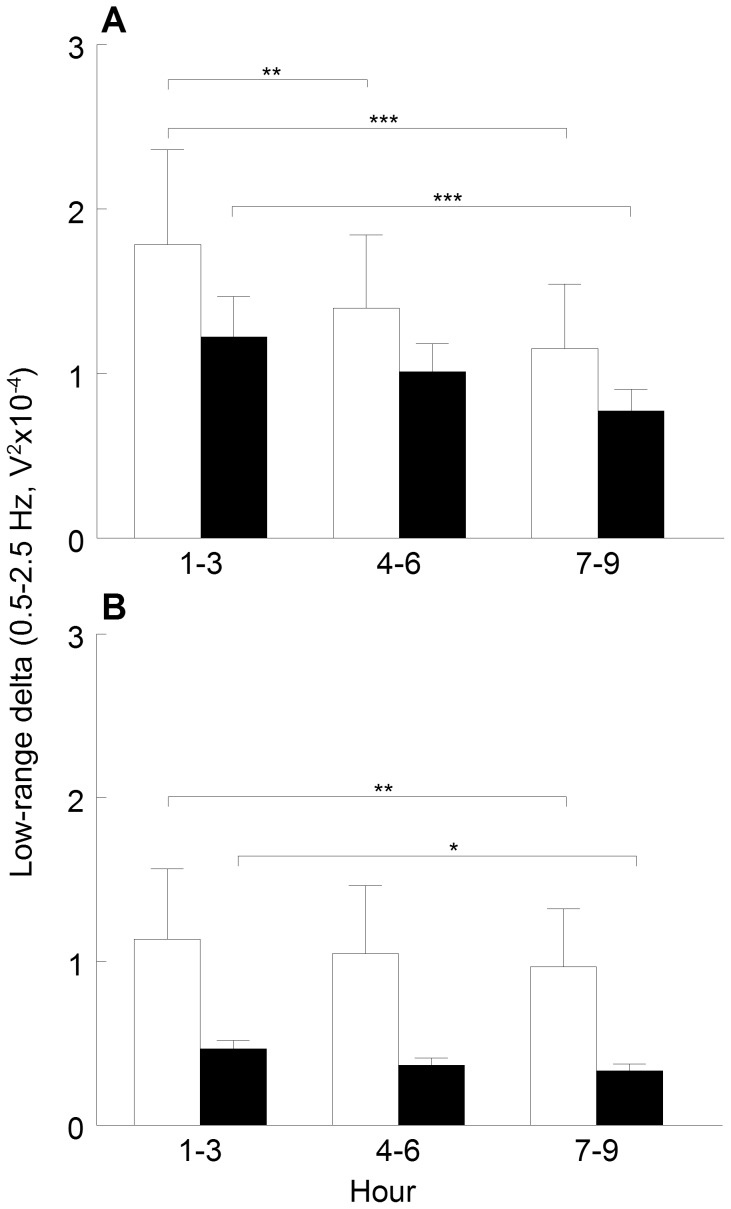
Sleep homeostasis. (A) pre and (B) post exposure to chronic mild stress. Mean values and SEM of EEG power within low-range delta band during slow wave sleep computed for 3 h blocks after lights on at 07∶00 A.M., in brief (white bars) and long maternally separated rats (black bars). Statistical analyses: hour effect pre (F_(2,20)_ = 22.2, *p*<0.001) and post (F_(2,24)_ = 8.8, *p*<0.01); recording day effect in h 1–3 (F_(1,10)_ = 27.0, *p*<0.001), h 4–6 (F_(1,12)_ = 18.2, *p*<0.01), and h 7–9 (F_(1,11)_ = 12.7, *p*<0.01).* *p*<0.05,** *p*<0.01,*** *p*<0.001.

Homeostatic sleep pressure, measured by the EEG power in the low-range delta band (0.5–2.5 Hz) was examined across hours spent in SWS during the sleep period (12 h inactive phase) (Figure 5). Descriptively, the homeostatic sleep pressure was lower in the LMS offspring compared to BMS both pre and post CMS, evident across the hours spent in SWS. However, these group differences were not statistically significant, either at time h 1 (first hour after lights on at 07∶00 A.M.; d =  −0.2), or at any of the 3 h bins (d≥−0.5).

Pre CMS, BMS offspring demonstrated a typical reduction in the sleep pressure throughout the sleep period. In LMS offspring a decreased sleep pressure was significant first after 7–9 hours into the sleep period (see Figure 5A).

Exposure to CMS decreased and flattened the curves of the low range power density throughout the sleep period (Figure 5B) equally in both groups. In LMS as well as BMS offspring, the sleep pressure was significantly reduced first after 7–9 hours of SWS.

### Sleep and Wakefulness

Mean values and results from statistical analyses for all parameters pre and post CMS are presented in Table 2. Pre CMS the two groups only differed in the quality of SWS. The LMS group spent more time in SWS2 and less in SWS1. After exposure to the CMS protocol, the LMS group spent more time in sleep, had more REM sleep episodes and higher percentage of NREM episodes ending in REM sleep compared to the BMS group. The longest episode duration of wakefulness was increased in the BMS group. CMS equally affected other sleep parameters in all offspring.

**Table 2:** Sleep parameters pre and post chronic mild stress (CMS) exposure.

		Pre CMS				Post CMS			
		LMS	BMS	*p*	d	LMS	BMS	*p*	d
	Total sleep time	555.31 (7.20)	541.00 (5.32)	ns	0.85	534.87 (7.35)*	505.49 (9.51)*	<0.05	1.32
Sleep stages	Wakefulness	158.39 (6.70)	172.90 (4.56)	ns	−0.97	183.06 (6.00)*	214.51 (9.51)***	<0.001	−1.53
	SWS1	317.73 (13.55)	348.35 (21.42)	<0.01	−0.92	363.96 (6.80)***	351.71 (14.61)	ns	0.43
	SWS2	129.74 (12.80)	84.28 (13.31)	<0.001	1.37	69.96 (4.07)***	53.10 (10.30)*	ns	0.94
	REM sleep	107.81 (3.89)	104.13 (4.35)	ns	0.33	100.96 (5.47)	82.83 (6.10)	ns	1.18
NREM sleep (SWS1+ SWS2) characterization	Number of episodes	776.43 (99.95)	781.67 (57.66)	ns	−0.07	618.14 (71.98)**	683.17 (145.56)*	ns	−0.60
REM sleep characterization	Percentage of NREM episodes ending inREM sleep	7.56 (1.07)	6.28 (1.07)	ns	0.68	8.92 (1.49)	6.11 (1.98)	<0.05	1.62
	Number of episodes	57.17 (2.41)	54.29 (3.63)	ns	0.37	51.50 (2.01)*	41.86 (4.04)***	0.056	1.24
	Mean episode duration	1.82 (0.07)	1.87 (0.10)	ns	−0.20	1.79 (0.05)	1.83 (0.11)	ns	−0.17
Wakefulness characterization	Number of episodes	104.14 (5.95)	107.14 (7.22)	ns	−0.17	106.14 (5.57)	112.43 (10.30)	ns	−0.30
	Mean episode duration	1.54 (0.07)	1.66 (0.12)	ns	−0.48	1.76 (0.12)	2.00 (0.18)	ns	−0.59
	Longest episode duration	32.57 (2.86)	36.88 (2.62)	ns	−0.59	30.93 (2.29)	39.98 (3.62)	<0.05	−1.16

All values are in minutes, except percentage and number of episodes; mean (SEM). LMS = long maternal separation, BMS = brief maternal separation.

Statistical analyses: Total sleep time: main effect of group and recording day (F’s_(1,12)_≥6.0, *p*<0.05); Sleep stages: group×sleep stage and group×sleep stage×recording day (F’s_(3,33)_≥3.1, *p*<0.05); Number of NREM sleep episodes: recording day (F_(1,11)_ = 17.2, *p*<0.01); percentage of NREM episodes ending in REM sleep: group×recording day (F_(1,11)_ = 9.6, *p*<0.05); Number of REM sleep episodes: group×recording day (F_(1,11)_ = 5.0, *p*<0.05); Mean duration of wakefulness episodes: recording day (F_(1,12)_ = 7.4, *p*<0.05); Longest duration of wakefulness episode: group (F_(1,12)_ = 6.1, *p*<0.05). Between group differences are shown by *P-*values and Cohen’s d effect sizes. Within group differences are denoted with*<0.05;**<0.01;***<0.001.

### Quality of SWS Differed as a Consequence of Maternal Separation

LMS offspring showed more SWS2 and less SWS1 compared to BMS. Statistical comparison did not show a difference between LMS and BMS offspring in total sleep time (555 min vs 541 min) or wakefulness (158 min vs 173 min) despite large effect sizes (d = 0.85 and d = −0.97, respectively), see Table 2. The two groups showed equal number of episodes of NREM sleep, REM sleep and wakefulness as well as duration of both REM sleep and wakefulness episodes. Time in REM sleep and percentage of NREM episodes ending in REM sleep were also similar between groups.

### CMS Increased Total Sleep Time in LMS Offspring

Exposure to four weeks of CMS similarly affected several sleep parameters in both groups. See Table 2. Total sleep time was reduced, wakefulness increased, SWS2 decreased and there were fewer NREM sleep and REM sleep episodes. SWS1 increased in the LMS offspring, while it was not affected in the BMS offspring. The BMS group showed an increased mean duration of wakefulness episodes. This change was not observed in the LMS group. In the LMS group, the percentage of NREM episodes ending in REM sleep was increased by CMS. However the amount of REM sleep was not affected by CMS in either group.

Compared to BMS, the LMS group spent more time asleep, though they did not show significantly higher amount of any particular sleep stage. Moreover, the LMS offspring were less awake than BMS (who showed longer duration of wakefulness episodes), showed higher percentage of NREM episodes ending in REM sleep as well a strong trend towards more REM sleep episodes (p = 0.056, d = 1.24).

## Discussion

This paper presents findings on EEG changes in adult rats after exposure to early life stress. The results indicate that long maternal separation affects the brain in lasting ways. Compared to brief maternal separation, LMS offspring showed a sleep-wake nonspecific reduction in adult brain activity at the frontal EEG derivation. Moreover, the sleep pressure was more slowly reduced and the quality of SWS differed from the BMS group. Exposure to chronic stress as adults led to lower brain activity in both groups. LMS offspring showed longer total sleep time, increased number of REM sleep episodes and percentage of NREM episodes ending in REM sleep compared to BMS offspring, while chronic stress equally affected the other sleep parameters and flattened the sleep homeostasis curves in all offspring.

Investigation of EEG power in both wakefulness and in the different sleep stages revealed a reduced EEG power in the LMS group compared to BMS. The finding was consistent across all frequencies, particularly in the low oscillating frequencies during SWS as well as the higher frequencies during REM sleep and wakefulness. Moreover, chronic mild stress showed a pronounced effect on EEG power. Independently of the dam-pup separation time, CMS reduced EEG power in all offspring. Hence, these results add significant findings to the literature of neuronal changes after long maternal separation and low maternal care [21]–[30], [39]. The present data in terms of lowered power of the wakefulness-characteristic EEG frequencies are reminiscent of findings of reduced EEG power of the higher frequencies during wakefulness in adult humans with a history of early life stress, and in post-institutionalized children [1], [2]. Interestingly, the LMS offspring showed a lower EEG power mainly in the frontal derivation compared to the BMS animals. Since all animals in our study were of the same age (120 days) at the time of recording, the lower frontal EEG power in LMS animals may reflect possibly neurodegenerative changes in frontal cortical structures. After exposure to CMS, the EEG power of the gamma frequency in the frontal-parietal derivation was lower in LMS compared to BMS offspring. This result may parallel the clinical finding of stress-altered cortical processing in the posterior regions after presentation of unpleasant pictures in victims of negative childhood experiences [5].

Computer analysis of EEG frequencies when awake or asleep provides a description of summed electrical potentials generated in the cerebral cortex in response to various inputs including those from deeper parts of the brain. The EEG power is directly correlated with thickness of the cerebral cortex and reflects its maturation [40]. In rats, a normal development of EEG rhythms continues until weaning at 3 weeks of age. Thereafter the EEG power increases and the characteristic features for each sleep stage appear [41]. Active maternal care behaviours such as licking and grooming and arched back nursing are recognised to be important factors in brain development [42]. Maternal separations of duration beyond what is considered to be natural for rats may therefore cause deviation from the normal development of EEG rhythms. Disrupting the mother-infant environment has been reported to affect neurogenesis, markers of neural plasticity, synaptic pruning, brain glia, and development of monoaminergic fiber systems and hippocampus, both in rodents and non-human primates [20], [22], [23], [27], [29], [39], [43]–[45]. Such neuronal changes could contribute to a detectible lowering of brain activation. The present study gives no further evidence of what kind of neuronal changes are responsible for the dampened EEG power after early life stress in rats. However a decrease in the EEG power may reflect a decrease in the *amplitude* or the *number* of these waves, or both [46].

Our data suggest that early life stress may affect sleep homeostasis. BMS offspring showed a typical and consistent reduction in sleep pressure from the first hours of the sleep period throughout the 12 h sleep time, whereas LMS offspring showed a slower homeostatic reduction, first significantly evident 7–9 hours into their sleep. In all mammals, the high slow wave activity in the beginning of the sleep period reflects the need for sleep that is built up during wakefulness. The sleep need decreases progressively during sleep and reaches low levels in late sleep [18], [19]. The precise underlying mechanisms still need to be clarified. However, slow waves do reflect the synchronous firing of large groups of cortical neurons coordinated by underlying slow oscillations [47]. A recent computer model of activity in the homeostatic network suggests that quantity of EEG power in the delta band is directly related to synaptic strength [48].

Although sleep disturbances after negative childhood experiences are widespread, we are not aware of studies investigating if sleep homeostasis is impaired. It has been suggested that the level of SWA during sleep may be a function of synaptic efficacy on network synchronization [6], [49]. If early life stress does influence the brain in terms of processing high-amplitude slow waves sufficiently, sleep homeostasis might be weakened through either impaired synaptic activation during wakefulness and/or reduced synaptic downscaling during sleep. Another possible explanation of decreased slow wave activity is disturbed sleep. Insomnia, increased nightly awakenings and restless sleep are often reported in humans with a history of early life stress [8]–[10]. Our data on sleep architecture do not show more fragmented sleep or poorer quality of SWS in the LMS offspring. In contrast, their SWS consisted more of the deep SWS2 compared to BMS group. More deep sleep was found in spite of lower delta power in the LMS group. Such an outcome is possible since scoring of deep sleep in rats depends only on the amount of the delta waves and does not require a minimum peak-to-peak amplitude. We believe that lower delta power but more SWS2 reflects impairment in the quality of deep sleep. On the other hand, an increased synaptic activation during wakefulness is tied to homeostatic regulation of slow wave activity [6], [49]. Therefore any sleep during the LMS offspring’s active phase may decrease their homeostatic sleep drive. We did not investigate sleep and wakefulness distribution during the offspring’s active phase. However, we observed that levels of locomotor activity preceding the sleep period were equal in the two groups, suggesting no increase in active phase sleep in LMS offspring (unpublished data).

Impaired homeostatic sleep regulation in depression was proposed by Borbely, a hypothesis derived from the two-process model of sleep regulation [14]. Compared to healthy controls, depressed patients show a slower homeostatic reduction of delta activity across time spent in NREM sleep [14], [15], [50]. A weakened homeostatic regulation is also reported in a mouse model of depression, where no typical increase in delta power after 6 h sleep deprivation was observed in a genetic line of helpless female mice [51]. Based on our data, early life stress may impair homeostatic sleep regulation by diminishing slow wave quality.

Independently of the early environmental condition, exposure to CMS reduced sleep quality (less deep sleep) and sleep quantity (shorter total sleep time, more wakefulness), changes typically reported after CMS in animal facility reared rats [33], [34]. The longer total sleep time in LMS offspring was also present after CMS. Moreover, compared to the BMS group, LMS were less awake and their SWS consisted more of the SWS2. The latter finding was non-significant but showed a large effect size (d = 0.94). Additionally, CMS flattened the curve of homeostatic sleep pressure in both groups. We are aware of only one previous study combining early and later life stress examining sleep changes. Here, an acute adult stressor (1 h cold stress) increased SWS and REM sleep in both BMS and LMS offspring [13]. In the present study chronic stressors affected REM sleep in the LMS offspring. Compared to BMS offspring, CMS resulted in 22% more REM sleep, an increase in percentage of NREM episodes ending in REM sleep and more REM sleep episodes. This may indicate an increased REM sleep pressure after early and later life stress, although the mean duration of REM sleep episodes was not increased. Similar REM sleep alterations have been described in a model of depression with high stress reactive mice [52]. Although clinical sleep EEG recording studies are needed, there is strong evidence that exposure to early life stress can decrease sleep quality, increase nightly awakenings and nightmare related distress, also lasting into adulthood [8]–[10], [53].

One limitation of this study was the small sample size. We identified a number of changes after LMS, although the small sample size limits our ability to reach definitive conclusions regarding the impact of early life stress on EEG power, sleep homeostasis regulation and sleep disturbances. To ensure the impact of our data, we used Cohen’s d to estimate the effect sizes. Effect sizes objectively measure the importance of an effect, independently of the sample size. Hence, it adds to the test statistics by reflecting what is going on in the data set. Consequently, the effect sizes of characteristic EEG frequencies for the different sleep stages and wakefulness reflected that LMS offspring showed lower EEG power compared to BMS offspring. Animal research demands working with low n’s. We therefore believe that results showing large effect size, albeit not reaching a statistical significance, should be reported. The homogeneity of the sample size was tested with Levene’s test. In some cases there was a significant heterogeneity for the post CMS values. However, when the sample size is small such a test is quite sensitive to even small differences. On the other hand, since the sample size in the two groups is comparable we believe that the differences in population variances have small consequence for conclusion derived from the test [54].

Secondly, we were not able to provide information on sleep onset latency, REM sleep latency or sleep efficiency as we chose not to disturb the animals during the continuous telemetric recording. Investigation of those parameters could have provided more information on sleep architecture.

### Conclusion

The present study shows that manipulating a normal dam-pup interaction in a controlled laboratory setting provokes a change in the offspring’s adult brain functioning. Long maternal separation induced a sleep-wake nonspecific reduction at the frontal EEG derivation, a slower reduction in sleep pressure and different SWS quality as compared to brief maternal separation. Moreover, the present findings support the notion that the response to stressful life events in adulthood is modulated by early life experience. Chronic exposure to mild stressors affected sleep differently in the two groups of maternal separation.
